# Laparoscopic cholecystectomy with laparoscopy-assisted transgastric rendezvous ERCP in gastric bypass patients

**DOI:** 10.1007/s00464-026-12704-w

**Published:** 2026-03-12

**Authors:** Sofia Liljegard, Per-Anders Larsson, Erik Haraldsson

**Affiliations:** 1 Department of Surgery, Institute of Clinical Sciences, Sahlgrenska Academy, University of Gothenburg, Gothenburg, Sweden; 2 Department of Research, Development and Innovation, Skaraborg Hospital, Skövde, Region Västra Götaland Sweden; 3 Department of Surgery, Skaraborg Hospital, Skövde, Region Västra Götaland Sweden; 4 Department of Surgery, Sahlgrenska University Hospital, Gothenburg, Region Västra Götaland Sweden

**Keywords:** Rendezvous ERCP, Gastric bypass, Transgastric, CBDS, Choledocholithiasis, Cholecystectomy

## Abstract

**Background:**

Laparoscopy-assisted transgastric rendezvous ERCP (LAERCP) is a perioperative treatment option for common bile duct stones (CBDS) in Roux-en-Y gastric bypass (RYGB) patients. Although rendezvous ERCP (RV-ERCP) is a safe treatment for CBDS, comparative data with LAERCP in larger cohorts are lacking. This study compares outcomes of laparoscopic cholecystectomy (LC) and LAERCP in RYGB patients with LC and rendezvous ERCP (RV-ERCP) in patients with unaltered anatomy, using an extensive validated national registry.

**Methods:**

A retrospective study on prospectively collected nationwide cohort data from the Swedish Registry of Gallstone Surgery and ERCP (GallRiks), including all patients from September 2016 to June 2021 who underwent LC with same-day rendezvous ERCP. Patients with prior RYGB (RYGB group) were compared to those without previous upper abdominal surgery (non-RYGB group). Outcome measures was therapeutic success, peri- and postoperative adverse events, procedural time and readmissions.

**Results:**

Seventy RYGB and 4342 non-RYGB patients were identified. CBDS were detected in 60 and 3067 patients, respectively. Therapeutic success was 100% in the RYGB group versus 91.4% in the non-RYGB group (*p* = 0.018). Perioperative adverse events occurred in 8.8% and 2.3% of cases (*p* < 0.001), but none in the RYGB group had postoperative consequences. Postoperative adverse events, antibiotic use and readmissions were similar. Median procedural time (180 vs. 131 min, *p* < 0.001) and hospital stay (2 vs. 1 days, *p* < 0.001) were longer for the RYGB group. No mortality occurred in either group.

**Conclusions:**

Concomitant rendezvous LAERCP during LC is a safe and effective method for managing CBDS after RYGB. Despite a higher rate of perioperative events, outcomes were favorable relative to previously published data for non-concomitant LAERCP. These findings support LAERCP with rendezvous technique as the standard of care for RYGB patients in Sweden and provide registry-based evidence to inform future international guidelines.

Common bile duct stones (CBDS) are frequently found among patients in Sweden undergoing cholecystectomy [1]. Symptomatic CBDS left untreated are associated with severe complications such as obstructive jaundice, cholangitis, and acute pancreatitis [1]. While the recommended treatment for acute cholecystitis is laparoscopic cholecystectomy, there is no standard management of CBDS. Possible treatment options are, among others, pre-, peri- or postoperative endoscopic retrograde cholangiopancreatography (ERCP) [2, 3]. In Sweden, a laparoscopic cholecystectomy and intraoperative ERCP with rendezvous technique (RV-ERCP) has become the most common treatment option for CBDS [2, 3]. Internationally, it remains more common to treat patients with CBDS through a two-staged procedure, wherein patients undergo ERCP and cholecystectomy separately [4].

The European Association for Endoscopic Surgery (EAES) recommends intraoperative ERCP for the management of CBDS, whenever feasible. In Sweden, many surgeons are traditionally trained to perform ERCP, which removes the need to coordinate multiple specialties in the operating room. While the EAES recognizes the challenges that many hospitals face in aligning surgical and endoscopic teams, it simultaneously advocates for the elimination of these logistical barriers to enable more frequent single-session procedures [5].

## Common bile duct stones in Roux-en-Y gastric bypass (RYGB) patients

After laparoscopic gastric bypass, there is an elevated risk of CBDS due to the rapid weight loss in combination with varied gallbladder function [6–8]. RYGB patients also have an increased risk of postoperative complications after a cholecystectomy [9]. The altered RYGB anatomy, where there is no direct passage to the duodenum, limits the possibilities to treat CBDS and perform a regular ERCP. Safe handling of CBDS in RYGB patients remains a matter of discussion and establishing a reliable and standardized method for stone clearance in this population is essential.

## Laparoscopy-assisted transgastric rendezvous ERCP (LAERCP)

Since the 1990s, numerous treatment options for CBDS in RYGB patients have been described and compared in previous studies [10, 11]. Other than laparoscopic removal, these methods include, for example, different endoscopic and radiological techniques in combination, open transjejunal enteroscopy, transgastric access via endoscopic ultrasound (EUS), radiological percutaneous intervention, and combined surgical and endoscopic techniques [10, 12, 13]. From a global perspective, the method of choice for treating these patients is not determined by professional consensus but rather by the treating centre's level of competence and available technical solutions.

A detailed review or comparison of previously mentioned methods is beyond the scope of this article, which instead focuses on laparoscopy-assisted transgastric rendezvous ERCP (LAERCP) for managing CBDS in RYGB patients. This is a technique used during laparoscopic cholecystectomy, in which a surgical trocar is inserted into the gastric remnant, through which ERCP is performed using the so-called rendezvous technique, passing a guidewire through the cystic duct into the duodenal lumen. Studies comparing the laparoscopy-assisted transgastric ERCP approach, without the rendezvous technique, to other methods of managing CBDS have demonstrated a successful papillary cannulation and intervention rate exceeding 90% [10, 14], as well as favorable long-term outcomes [15]. A systematic review and meta-analysis conclude a 93% success rate for the LAERCP [16], but the included studies did not entail the rendezvous technique. In Sweden, the rendezvous LAERCP is the most frequently used technique for RYGB patients with CBDS.

We previously performed a small clinical retrospective study, where we compared rendezvous LAERCP in RYGB patients to RV-ERCP in patients with unaltered anatomy [17], showing equivalent outcome for the rendezvous LAERCP group compared to regular RV-ERCP. The study was small, as are most studies on LAERCP [11, 18–20]. To date, we have not found any previous studies on a larger cohort comparing the outcomes of handling CBDS in RYGB patients with those of patients with unaltered anatomy using the rendezvous technique.

### Aim

The study aims to compare the outcomes of laparoscopic cholecystectomy with concomitant rendezvous LAERCP in RYGB patients with those of a group of patients with unaltered anatomy undergoing laparoscopic cholecystectomy with concomitant regular RV-ERCP in a large Swedish national registry cohort.

## Methods

This is a retrospective study on prospectively collected nationwide cohort data from the Swedish Registry of Gallstone Surgery and ERCP (GallRiks). GallRiks is a validated registry, shown to have 97% accuracy compared to medical records, and has included over 90% of all cholecystectomies and ERCPs performed in Sweden since 2009 [21].

Since September 2016, GallRiks has registered a variable showing previous RYGB surgery, therefore, the data from September 2016 to June 2021 were analyzed. All laparoscopic cholecystectomies with an ERCP procedure performed during the same day using the rendezvous technique were extracted from the registry. The patients were then divided into those who had previous RYGB surgery and compared to patients with no previous upper abdominal surgery. Entries with missing data or having other previous upper abdominal surgery were excluded. The register has no variable for LAERCP but instead has one that specifies how the duodenoscope was inserted; hence, for RYGB patients, the duodenoscope had to be inserted through a laparoscopically created gastrostomy to be included in the RYGB group.

The primary outcome measure was therapeutic success (defined as successful stone clearance if stones were found during ERCP). Secondary outcome measures were procedural time, length of hospital stay, postoperative antibiotics, readmissions within 30 days, and peri- or postoperative adverse events: including bleeding, perforation, post-ERCP pancreatitis, and cholangitis.

There is currently no nationally mandated standardized technical protocol for LAERCP or RV-ERCP in Sweden. Consequently, minor variations inoperative and endoscopic technique may exist between centers. The following descriptions therefore reflect the general procedural principles applied, allowing reproducibility while acknowledging heterogeneity in clinical practice.

### RV-ERCP procedure

The laparoscopic cholecystectomy is performed with regular four port placement. Midways in the procedure, after performing the cholangiography, a guidewire is placed through the cystic duct into the duodenum. Here the ERCP procedure commences with exsufflation of laparoscopy gas and introduction of a duodenoscope per-orally down to the duodenum. The guidewire is then endoscopically grasped with a polypectomy snare through the duodenoscope and retrieved out through the mouth. A sphincterotome is then inserted over the guidewire and ensure safe cannulation of the common bile duct for stone removal while preventing pancreatic cannulation. After finished ERCP procedure the remaining laparoscopic cholecystectomy is carried out.

### LAERCP procedure

The laparoscopic cholecystectomy is performed in the same manner as in a RV-ERCP. After intraoperative cholangiography and placing the guidewire into the duodenum, the procedure continues with mobilization of the gastric remnant, enough to enable the anterior part of the greater curvature to reach the anterior abdominal wall. An additional 15 mm port is placed in the upper left quadrant of the abdomen. A purse-string suture is applied to the gastric remnant and a gastrotomy is made, where the 15 mm port is inserted and a purse-string is used to lift the gastric remnant to the abdominal wall and tied to secure it to the port. After exsufflation of laparoscopy gas, the ERCP is then performed through the 15 mm port in the same manner as in a regular RV-ERCP. After the ERCP procedure, the gastric remnant is loosened from the 15 mm port and the gastrotomy incision closed through either sutures or linear stapler. The remaining part of the laparoscopic cholecystectomy is thereafter performed and concludes the procedure.

### Statistical analysis

Continuous data were described in median with 25th and 75th percentiles. Categorical data were expressed in frequencies and percentages. Comparisons between the groups with respect to categorical variables were done by the Chi-square test or the Fisher’s exact test. Discrete data and continuous variables were compared between the groups by the Mann–Whitney test and a *p* value < 0.05 was considered statistically significant. Patients with missing data were excluded. IBM SPSS version 28 was used for all data analysis (IBM Corp., 2020, IBM, Armonk, NY, USA).

## Results

**Fig. 1:**
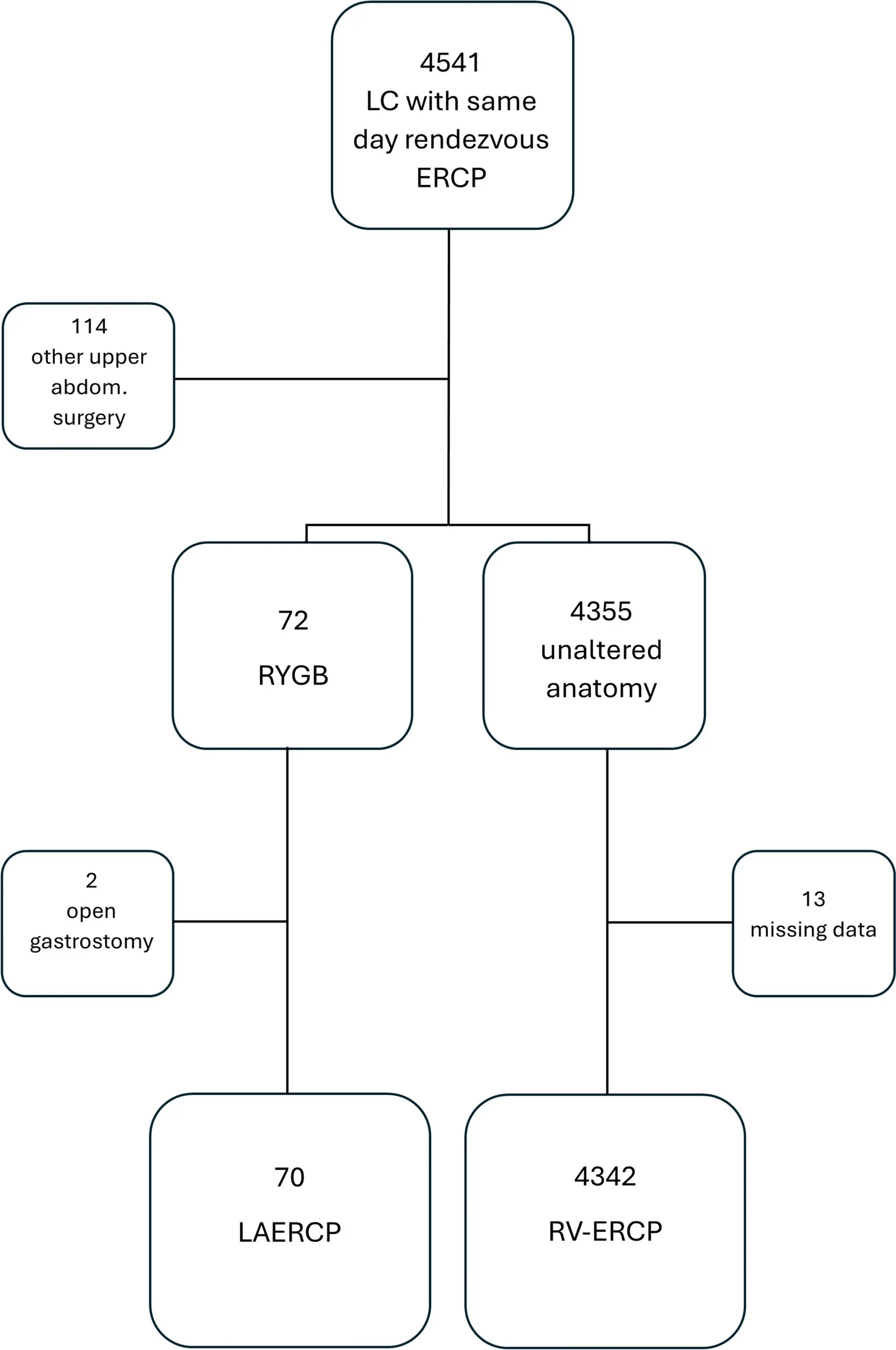
Cohort inclusion flowchart. *LC* Laparoscopic cholecystectomy, *RYGB* Roux-en-Y gastric bypass, *LAERCP* Laparoscopy-assisted transgastric rendezvous ERCP, *RV-ERCP* Rendezvous ERCP

There were 70 LAERCP (RYGB group) and 4342 regular RV-ERCP (non-RYGB group) procedures registered during the study period (Fig. 1). There were more women in the RYGB group compared to the non-RYGB group (81% vs 60%, *p* < 0.001). Out of 4541 patients that had a laparoscopic cholecystectomy with same day rendezvous ERCP, 13 were excluded for missing data in the registry, 114 had other upper abdominal surgery, and two were registered as having the duodenoscope inserted through an open gastrostomy.

During the study period, the 70 LAERCP procedures in the RYGB group were performed across 27 centers, with center volumes ranging from one to 11 procedures (median two procedures per center). These procedures were carried out by 53 surgeons, each performing between one and eight procedures (median one per surgeon). In 29 cases, the laparoscopic cholecystectomy and rendezvous LAERCP were performed by the same surgeon. The 4342 RV-ERCP procedures in the non-RYGB group were performed across 54 centers, with center volumes ranging from one to 411 procedures (median 59 procedures). A total of 825 surgeons contributed to these cases, each performing between one and 74 procedures (median three per surgeon).

The RYGB group was younger (median age 53 vs 56 *p* = 0.012) and had a higher body mass index (BMI) (median 31 vs 28 *p* = 0.006) than the non-RYGB group. There was no significant difference between the groups in preoperative health status, ASA (American Society of Anaesthesiologists) classification, or preoperative C-reactive protein (CRP) level (Table 1). The most common indication for ERCP registered in both groups was suspected or known CBDS (68 of 70 patients vs 4060 of 4342 patients). Other indications, much less frequent, included perioperative anatomical mapping, cholangitis or sepsis, jaundice or test results indicating biliary obstruction and acute pancreatitis, with no significant differences between the groups.

**Table 1:** Patient demographics comparison between laparoscopy-assisted transgastric rendezvous ERCP (RYGB group) and rendezvous ERCP (non-RYGB group)

	RYGB *n* = 70	Non-RYGB *n* = 4342	*P* value
Age (year)^a^	53 (41–61)	56 (39–71)	0.012
Gender			
Female	59 (84%)	2616 (60%)	< 0.001
Male	11 (16%)	1726 (40%)	
ASA			0.519
I	17 (24%)	1433 (33%)	
II	44 (63%)	2154 (50%)	
III–IV	9 (13%)	755 (17%)	
BMI^a^	31 (26–35)	28 (25–32)	0.006
CRP^a^	108.5 (58–133)	92 (32–170)	0.642

^a^Described in median and percentiles P25–P75

**Fig. 2:**
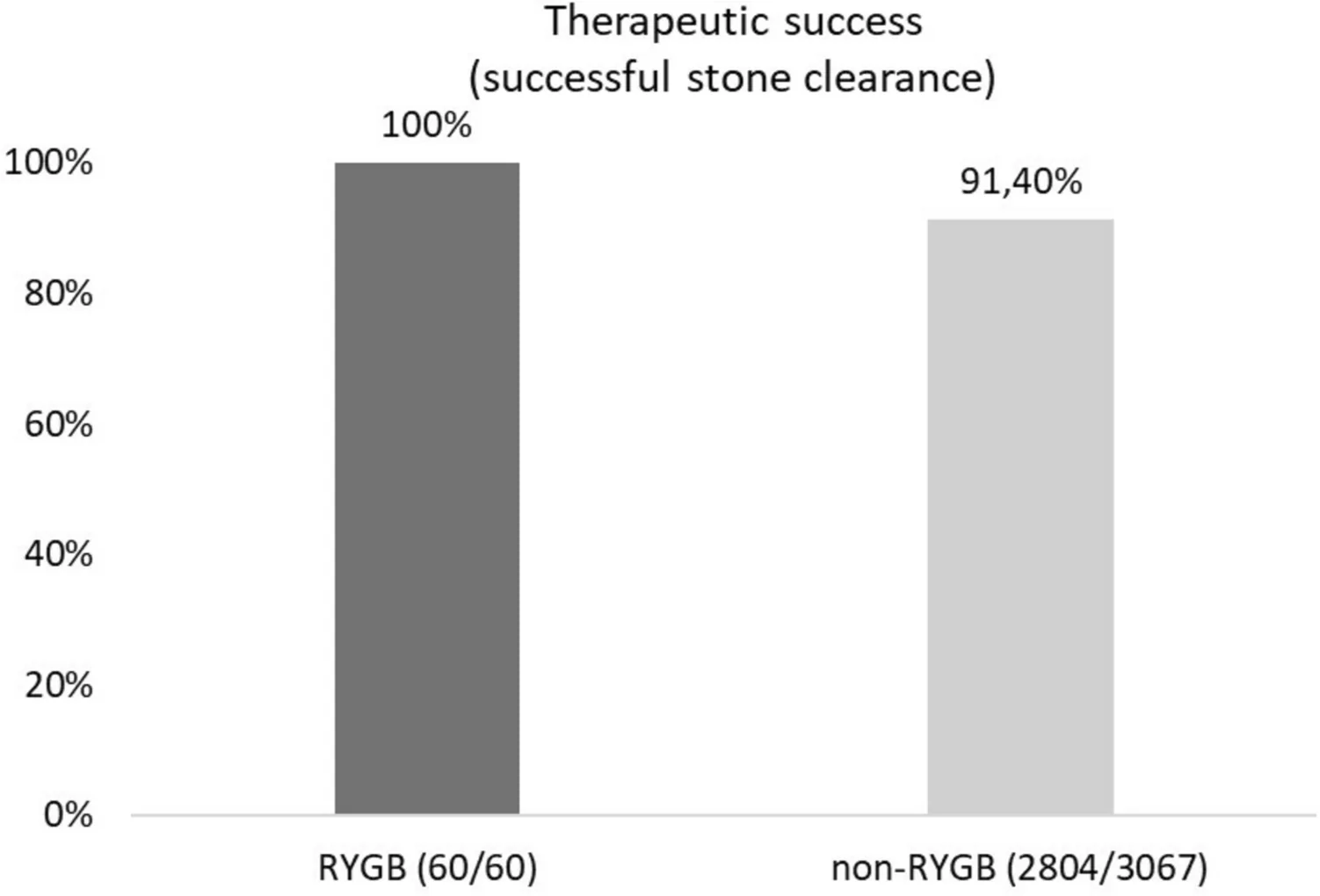
Illustrating therapeutic success as a percentage and the number of successful stone clearances in the number of patients with common bile duct stones

CBDS were found during ERCP in 60 patients in the RYGB group, compared to 3067 in the non-RYGB group. Among those, the RYGB group had 100% therapeutic success in stone clearance compared to 91.4% in the non-RYGB group (*p* = 0.018) (Table 2; Fig. 2). Patients with missing registrations for CBDS or registered with only sludge or pus were excluded from the calculations for successful stone clearance.

**Table 2:** Outcome comparison between laparoscopy-assisted transgastric rendezvous ERCP (RYGB group) and rendezvous ERCP (non-RYGB group)

	RYGB *n* = 70	Non-RYGB *n* = 4342	*P* value
Surgical data			
Emergency surgery^a^	53 (75.7%)	3146 (72.5%)	0.783
Surgery time (min)^b^	168.5 (120–242)	121 (88–168)	< 0.001
Successful stone clearance^c^	60/60 (100%)	2804/3067 (91.4%)	0.018
Perioperative adverse events^b^	6 (8.6%)	98 (2.3%)	< 0.001^e^
Postoperative data			
Postoperative antibiotics	5 (7.2%)	327 (7.6%)	0.911
Postoperative length of stay (day)^a^	2 (1–3)	1 (1–2)	< 0.001^d^
Postoperative adverse events	5 (7.2%)	541 (12.6%)	0.184^e^
Postoperative pancreatitis	0 (0%)	141 (3.2%)	

^a^Described in frequencies and percentages

^b^Described in median and percentiles P25–P75

^c^Out of patients with stones present

^d^Mann-Whitney test

^e^Fischer’s exact test

**Fig. 3:**
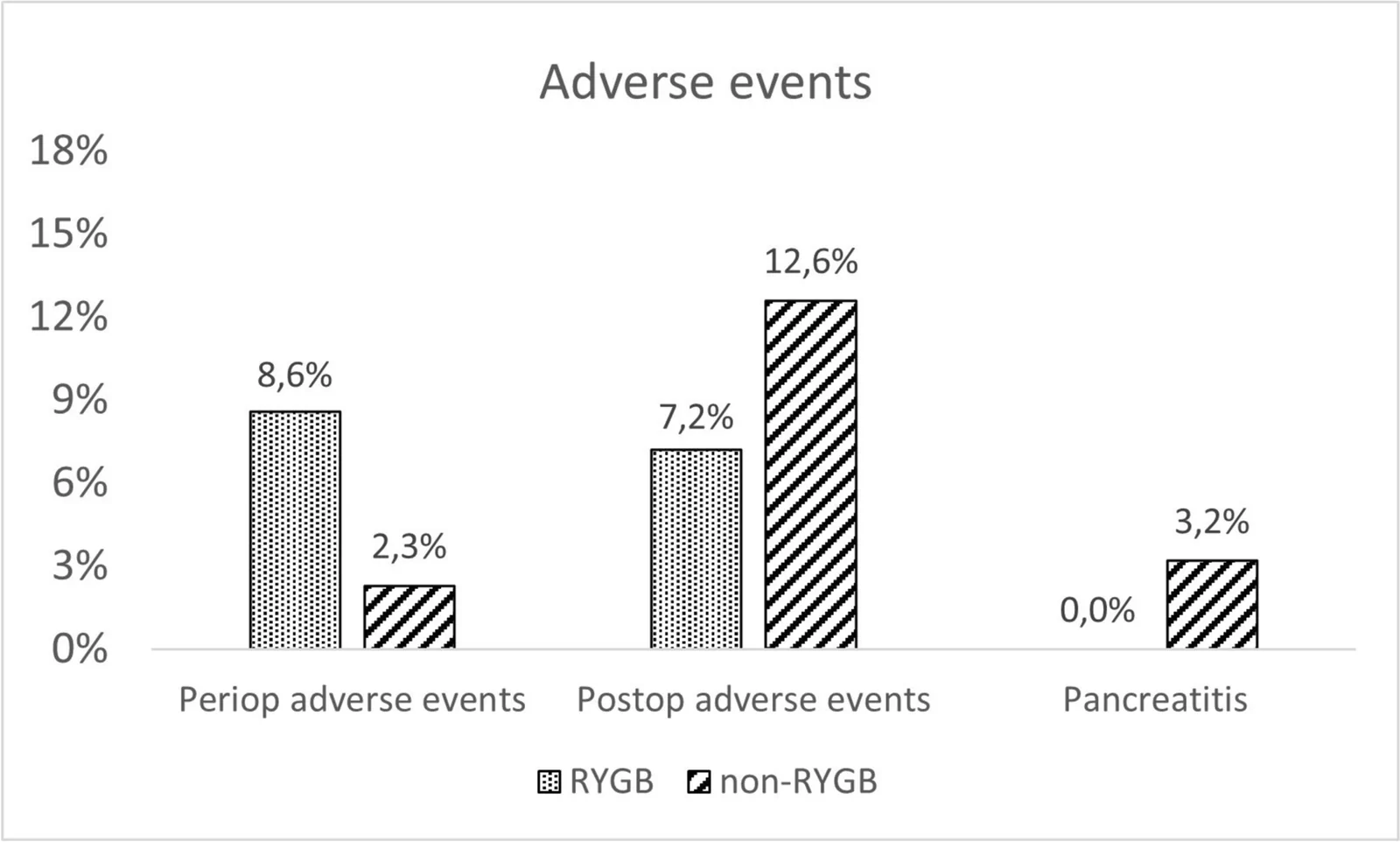
Illustrating the comparison of adverse events between groups

While there was no mortality in either group, six patients (8.6%) in the RYGB group and 98 patients (2.6%) in the non-RYGB group experienced an intraoperative adverse event (*p* < 0.001) (Fig. 3). At the same time, there was no significant difference in postoperative adverse events (RYGB 7.2% vs non-RYGB 12.6%, *p* = 0.184) (Table 2). Regarding perioperative adverse events in the RYGB group, four patients had perioperative bleeding, and two patients had no registered cause for the perioperative adverse event. In the RYGB group, no postoperative pancreatitis, cholangitis, blood transfusions, or thrombosis were reported. Two of the patients were reported as having had postoperative bleeding without transfusion, two had a postoperative infection, and one patient had no reported cause for the postoperative adverse event. Readmissions within 30 days were similar between the groups.

Postoperative antibiotics were administered in equal percentages in both groups. There was a significantly longer procedural time in the RYGB group (180 vs 131 min) (*p* < 0.001), and a longer postoperative length of hospital stay (median two vs one days) (*p* < 0.001). Procedures performed in an emergency setting were similar between the groups (76% vs. 73%, *p* = 0.783).

## Discussion

Adapting the rendezvous technique to treat patients with CDBS and altered anatomy after RYGB, by adding a laparoscopically created gastrostoma to introduce the duodenoscope, appears, in this national cohort to be as safe and feasible as in patients with naive anatomy.

This study analyses a large patient cohort from the Swedish National Quality Registry for Gallstone surgery and ERCP (GallRiks), not aiming to compare different methods of CBDS treatment within the RYGB group, but rather to assess whether the rendezvous technique, when performed concomitantly with cholecystectomy, is as safe and effective with altered anatomy after RYGB as it is in those with unaltered anatomy. In this small, but largest to date, study cohort there was 100% therapeutic success in the RYGB group regarding CBDS clearance and no postoperative pancreatitis, cholangitis, thrombosis, or bleeding in need of blood transfusions in the study group. Between the groups, there were minimal or no differences concerning the proportion of surgeries conducted in an emergency setting, preoperative CRP levels, postoperative antibiotic use, and readmission within 30 days. There was no mortality in any of the groups.

Although laparoscopic stone extractions, both transcystic or through a choledochotomy, are methods used in Sweden for RYGB patients requiring biliary clearance, LAERCP has grown to be the most common treatment option. While several alternatives are available, such as balloon-assisted enteroscopy ERCP or EUS-directed transgastric ERCP (EDGE) - current international guidelines do not take a definitive position regarding which method should be preferred in RYGB patients. The rendezvous technique is applicable in both elective and emergency settings. The patient selection should be based primarily on clinical condition whether the patient can safely be treated in a combined intervention. Our results, therefore, contribute necessary real-world evidence supporting the safety and efficacy of the most common approach in Sweden.

The success rate of rendezvous LAERCP in RYGB patients does not demonstrate any greater efficiency than RV-ERCP in non-RYGB patients. Instead, the extra procedures of the rendezvous LAERCP probably make the surgeon more inclined to ensure all CBDS are cleared, rather than leaving a stent or hope for the stones to pass spontaneously, avoiding the need to plan the patients for subsequent procedures.

Although GallRiks is a validated, large, Swedish national register that accurately records the surgical procedures performed, it does not capture the clinical rationale behind each procedural choice. Nevertheless, given that LAERCP is the most common method nationally for RYGB patients, it is reasonable to assume that most cases followed the current Swedish best practice.

Using intraoperative rendezvous ERCP to manage CBDS during laparoscopic cholecystectomy has previously been shown to have low post-ERCP pancreatitis frequencies [22]. Likewise, in the present study, the RYGB group had no reported cases of postoperative pancreatitis and the non-RYGB group also had a low postoperative pancreatitis rate (3.2%). This emphasizes the advantage of using the rendezvous technique to clear CBDS. Previous studies of LAERCP without using the rendezvous technique, show an average 7% postoperative pancreatitis frequency [16, 23]. The laparoscopy-assisted transgastric rendezvous ERCP technique used intraoperatively during laparoscopic cholecystectomy has, in this Swedish national cohort, been associated with better outcomes.

Six patients (8.6%) were reported to have suffered perioperative adverse events in the RYGB group, but non with persisting postoperative consequences. Although significantly more perioperative adverse events than in the non-RYGB group (2.3%), the results still compare favorably to previously reported data for LAERCP, with or without a concomitant laparoscopic cholecystectomy, where there have been reports of up to 19% perioperative adverse events [14, 16, 23]. Two of the six intraoperative adverse events in the RYGB group were recorded without a detailed cause. The registry provides a limited set of predefined options for intraoperative adverse events; accidental perforation of the gallbladder, bowel perforation, intraoperatively detected bile duct injury, cardiovascular complication, pulmonary complication, medical device related problems and bleeding requiring intervention. If an event doesn’t fit clearly within the available categories, the event can be registered without a specific cause. The absence of a specific category does not indicate absence of an adverse event but rather reflects limitations in the registry’s classification system.

The procedural time was significantly longer in the RYGB group than in the non-RYGB group, likely because the LAERCP procedure requires the insertion of an additional port in the gastric remnant. There may also be more time spent to resolving abdominal adhesions during gastric remnant mobilization, and it is common to inspect and close any open mesenteric slits if encountered.

The postoperative length of stay was significantly longer in the RYGB group (median two vs one days). The prolonged hospital stay could be anticipated, given the added trauma of the gastrotomy considering both the patient group condition as well as the surgeon’s concern not to overlook any possible complication.

There is a high acceptance of RV-ERCP in Sweden, which can be due to the frequent use of intraoperative cholangiography (IOC) to detect CBDS, and the fact that most of the intraoperative ERCPs are performed by an advanced endoscopy fellowship-trained surgeon [4]. This routine eliminates the need to coordinate two specialities in the operating room, thereby facilitating easy access to unplanned emergency ERCP as well as minimizing intraoperative changing times. The broad distribution of cases across centers and operators suggests that the safety and feasibility of the rendezvous approach depend not on individual high-volume expertise, but rather on access to appropriate competence and organizational support. National guidelines in both Sweden and Denmark recommend laparoscopic cholecystectomy and CBDS removal in a one-step procedure [24, 25]. This is in line with EAES recommendations to aim toward more intraoperative ERCP when common bile duct stones are present [5].

The retrospective design of this study is a weakness. As is the use of registry data, with missing entries and bias in the original selection of what treatment was chosen for which patient. To ensure consistent use of the RYGB variable, only entries between 2016 and 2021 were used. Although the registry data are comprehensive, some perioperative variables may not be fully captured. Nonetheless, the use of data from an extensive nationwide validated registry, providing a large study population, is a major strength of this study.

## Conclusion

Laparoscopy-assisted transgastric rendezvous ERCP during laparoscopic cholecystectomy is a safe and effective method for treating CBDS in patients with prior RYGB surgery, with outcomes comparable to standard rendezvous ERCP during laparoscopic cholecystectomy in patients with naive anatomy. Our findings indicate that the rendezvous technique can be safely applied in patients with complex altered anatomy such as after RYGB. The principal limitation to wider implementation is therefore not patient suitability, but access to appropriate surgical and endoscopic expertise and organizational capacity. The result of this study supports the continued use of LAERCP approach as the standard of care in Sweden and contribute valuable outcome data to an international context where definitive guideline recommendations remain lacking.
