# Correction: Laparoscopic cholecystectomy with laparoscopy-assisted transgastric rendezvous ERCP in gastric bypass patients

**DOI:** 10.1007/s00464-026-13399-9

**Published:** 2026-09-30

**Authors:** Sofia Liljegard, Per-Anders Larsson, Erik Haraldsson

**Affiliations:** 1 Department of Surgery, Institute of Clinical Sciences, Sahlgrenska Academy, University of Gothenburg, Gothenburg, Sweden; 2 Department of Research, Development and Innovation, Skaraborg Hospital, Skövde, Region Västra Götaland Sweden; 3 Department of Surgery, Skaraborg Hospital, Skövde, Region Västra Götaland Sweden; 4 Department of Surgery, Sahlgrenska University Hospital, Gothenburg, Region Västra Götaland Sweden

**Correction to: Surgical Endoscopy (2026) 40:4317–4325** **https://doi.org/10.1007/s00464-026-12704-w**

The original online version of this article was revised to correct the serial number of the institutional ethics approval documentation. The correct information is as follows:

The Swedish Ethical Review Authority, Serial Number 2021-06159-01, approved on December 15, 2021.

The original article has been corrected.

